# Loss of the *NKX3.1* tumorsuppressor promotes the *TMPRSS2-ERG* fusion gene expression in prostate cancer

**DOI:** 10.1186/1471-2407-14-16

**Published:** 2014-01-13

**Authors:** Rajesh Thangapazham, Francisco Saenz, Shilpa Katta, Ahmed A Mohamed, Shyh-Han Tan, Gyorgy Petrovics, Shiv Srivastava, Albert Dobi

**Affiliations:** 1 Center for Prostate Disease Research, Uniform Services University of the Health Sciences, 1530 East Jefferson Street, Rockville, Maryland 20852, USA

**Keywords:** Tumor suppressor, NKX3.1, Prostate, ERG, NFкB, Oncogene

## Abstract

**Background:**

In normal prostate epithelium the *TMPRSS2* gene encoding a type II serine protease is directly regulated by male hormones through the androgen receptor. In prostate cancer *ERG* protooncogene frequently gains hormonal control by seizing gene regulatory elements of *TMPRSS2* through genomic fusion events. Although, the androgenic activation of *TMPRSS2* gene has been established, little is known about other elements that may interact with *TMPRSS2* promoter sequences to modulate *ERG* expression in *TMPRSS2-ERG* gene fusion context.

**Methods:**

Comparative genomic analyses of the *TMPRSS2* promoter upstream sequences and pathway analyses were performed by the Genomatix Software. *NKX3.1* and *ERG* genes expressions were evaluated by immunoblot or by quantitative Real-Time PCR (qRT-PCR) assays in response to siRNA knockdown or heterologous expression. QRT-PCR assay was used for monitoring the gene expression levels of NKX3.1-regulated genes. Transcriptional regulatory function of NKX3.1 was assessed by luciferase assay. Recruitment of NKX3.1 to its cognate elements was monitored by Chromatin Immunoprecipitation assay.

**Results:**

Comparative analysis of the *TMPRSS2* promoter upstream sequences among different species revealed the conservation of binding sites for the androgen inducible *NKX3.1* tumor suppressor. Defects of *NKX3.1*, such as, allelic loss, haploinsufficiency, attenuated expression or decreased protein stability represent established pathways in prostate tumorigenesis. We found that NKX3.1 directly binds to *TMPRSS2* upstream sequences and negatively regulates the expression of the *ERG* protooncogene through the *TMPRSS2-ERG* gene fusion.

**Conclusions:**

These observations imply that the frequently noted loss-of-function of NKX3.1 cooperates with the activation of *TMPRSS2-ERG* fusions in prostate tumorigenesis.

## Background

Activation of the *ERG* oncogene [1] represents an early event in pre-neoplastic to neoplastic transition during prostate tumorigenesis [2-4]. Rearrangements between the androgen regulated *TMPRSS2* gene promoter and the ETS-related *ERG* gene result in *TMPRSS2-ERG* fusion transcripts that have been found in approximately half of prostate cancer cases in the Western world [5]. Fusion of other androgen regulated genes, such as, the prostein coding *SLC45A3*, prostate specific antigen homologue kallikrein 2 (*KLK2*) or the N-MYC downstream regulated gene 1 (*NDRG1*) contribute to *ERG* activation with lower frequencies [6]. At protein levels ERG is detected as a nearly uniformly overexpressed protein in over 60% of prostate cancer patients as revealed by the diagnostic evaluation of ERG oncoprotein detection in prostatic carcinoma [7,8].

Much has been learned about the androgenic regulation of *TMPRSS2* promoter [9-13] in prostate cancer. In contrast, other control elements of the *TMPRSS2* promoter are largely unexplored both in the wild type, as well as, in the *TMPRSS2-ERG* fusion genomic context. In the current study comparative analysis of *TMPRSS2* promoter upstream elements among different species revealed the presence of a conserved NKX3.1 binding site.

*NKX3.1* is a *bona fide* tumor suppressor gene with prostate-restricted expression [14]. Loss or decreases in NKX3.1 levels has been frequently observed in prostatic intraepithelial neoplasia and at the pre-neoplastic to neoplastic transformation stages of prostate cancer [15,16]. Loss of *Nkx3.1* cooperates with loss of *Pten* in engineered mouse models of prostate tumorigenesis [17,18]. Furthermore, Nkx3.1 defects cooperate with Pten-Akt pathways [19] and disrupt cellular response to DNA damage [20]. Nkx3.1 was also shown to oppose the transcription regulatory function of C-Myc [21] in mouse models. In prostate cancer cells *C-MYC* is activated by *ERG*[22-24]. A recent study has shown that ERG is a repressor of *NKX3.1* raising the possibility of a feed-forward circuit in prostate tumorigenesis [25]. Our observation of conserved NKX3.1 binding elements in the *TMPRSS2* promoter prompted us to examine the hypothesis that NKX3.1 is a repressor of *ERG* in the *TMPRSS2-ERG* fusion genomic context in prostate cancer.

## Results

### Identification of an NKX3.1 binding site within the *TMPRSS2* gene promoter upstream sequences

**Figure 1:**
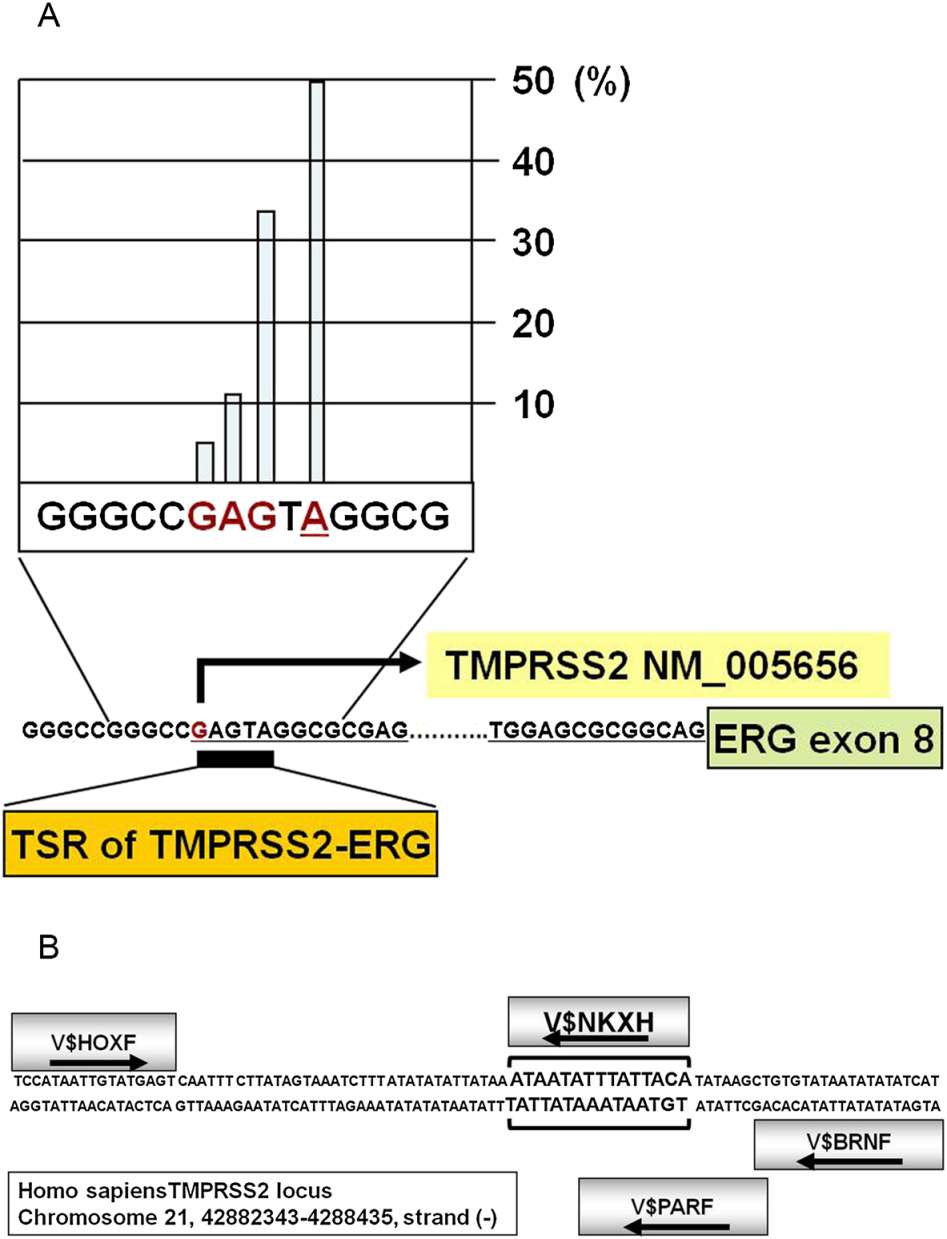
**Defining a conserved composite model for NKX3.1 binding within the *TMPRSS2 *gene promoter upstream sequences. (A)** Frequency of *TMPRSS2-ERG* transcript initiation sites within the *TMPRSS2* promoter transcriptional start region (TSR). **(B)** NKX3.1 model match within the human *TMPRSS2* promoter upstream region with conserved distance, positions and orientations (arrows) of transcription factor binding sites.

Within the *TMPRSS2* gene locus promoter downstream sequences beyond the +78 position of the first non-coding exon (NM_005656) frequently participate in genomic rearrangement events. These genomic rearrangements are characterized by the recurrent *TMPRSS2* (first non-coding exon:+78) [26] to *ERG* (exon 8 or Exon 9) [1,27,28] fusion junctions also known as fusion type “A” or “C”, respectively [11]. In this gene fusion event the *TMPRSS2* promoter-proximal and promoter upstream sequences are retained. Towards the bioinformatic analysis of *TMPRSS2-ERG* regulatory elements we mapped the transcription start sites (TSS) of *TMPRSS2* gene in *TMPRSS2-ERG* fusion harboring human prostate tumors. From a carefully characterized RNA pool of *ERG* expressing and *TMPRSS2-ERG* fusion harboring prostate tumors obtained from six radical prostatectomy specimens [29], cDNA molecules were generated and amplified using 5’ cap-specific forward primers and *ERG*-specific reverse primers. Amplicons were isolated and cloned. Individual clones (n = 20) were analyzed by DNA sequencing and the frequency of cap-tags were plotted on the transcription start region (TSR_200587) of the *TMPRSS2* gene (Figure 1A). The DNA sequence analysis revealed that the most frequent (50%) transcription start of *TMPRSS2-ERG* fusion transcripts is at +5, relative to the wild type *TMPRSS2* promoter +1 position. By confirming the TSS position we focused our investigation on the +78 to15,000 upstream regulatory region of the *TMPRSS2* gene on chromosome 21 (NCBI build 36.3) for further analyses. This genomic region encompasses upstream regulatory elements (-13.5 kb) shown to control cancer-associated expression of the ERG oncogene [30].

Comparative analysis of modular regulatory sequences of various species is a powerful approach for pinpointing functionally relevant regulatory elements [31-33]. We applied a computational approach (FrameWoker software, release 5.4.3.3) that has been shown to identify conserved orientation, relative position and relative distance of binding motif (matrix) clusters [34,35] also known as the “motif grammar” [36] using the Matrix Family Library Version 7.1. We have examined the -15,000;+78 bp regions of human, rhesus monkey, rat and mouse *TMPRSS2* gene promoter upstream sequences for the conservation of composite regulatory elements. Striking conservation of a composite model was noted in this analysis that was mapped to the human *TMPRSS2* -2350; -2258 sequences relative to the TSS. Within the composite model we have identified the vertebrate NKX3.1 matrix (V$NKXH) as the prostate-specific component of the model and putative binding site was termed as NKX3.1 binding site 1 (NBS1) (Figure 1B).

### NFкB-centered network of NKX3.1 target gene signatures

**Figure 2:**
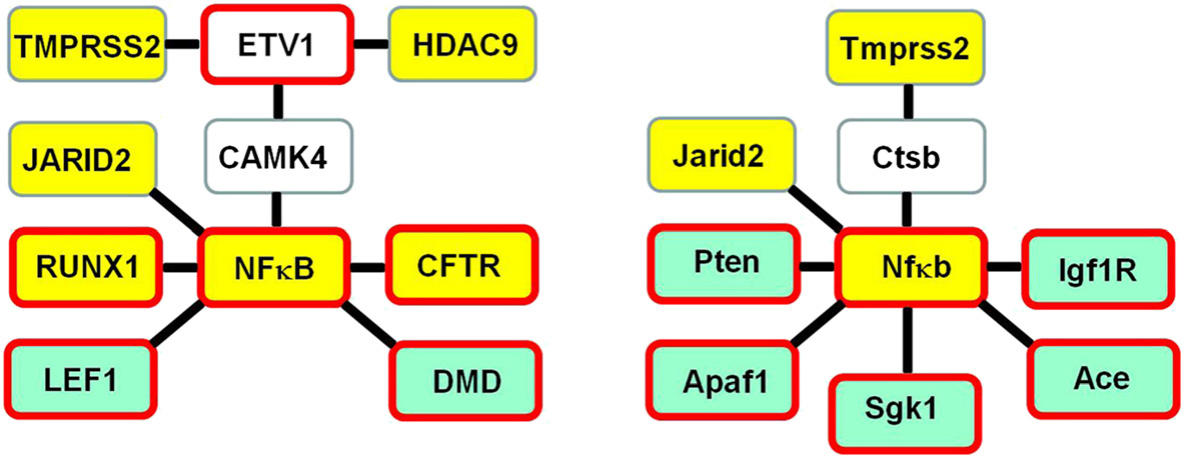
**Summary of NFкB centered NKX3.1 target gene signatures from *in silico *(left panel) and from the meta-analysis of *in vivo *data (right panel).** Experimentally validated human genes and their orthologues in mouse are highlighted in yellow. Secondary nodes representing genes with four or more functional connections are stemming from the central regulatory node (green boxes). Nodes with four or more functional connections are outlined by red. Connected genes are marked with white background color.

Utilizing this highly conserved model the entire human genome was searched for model matches (ModelInspector Release 5.6) to define gene loci potentially targeted by NKX3.1. After filtering for non-redundant, intronic, exonic and promoter model matches within gene loci of annotated genes, knowledge-based pathway analysis was performed using functional co-citation settings. The analysis revealed a network with NFкB in the central regulatory node (Additional file 1: Figure S1). As expected, searching of the entire human genome for this composite model precisely identified the *TMPRSS2* gene upstream -2350; -2258 sequences. In contrast, search of the dog, bovine, opossum and zebra fish genome failed to identify model matches within the *Tmprss2* loci of these species. In a meta-analysis approach we compared the comparative genome analysis-derived network to the signature of *Nkx3.1*-targeted genes defined by *in vivo* ChIP assay in a mouse model (Additional file 1: Figure S2) [21]. Strikingly similar NFкB-centered regulatory network was revealed by the analysis (Figure 2). NKX3.1 target genes within the compared datasets were enriched in functionally related genes. Moreover, the analysis highlighted orthologues of *TMPRSS2*, *JARID2* and the *NF*к*B* genes. The apparent similarity between these datasets has prompted us to examine the disease association of NKX3.1 target genes by gene ontology analyses. Enrichment of chromosome aberrations, inversion, breakage and associated diseases was revealed by the analysis (Table 1).

**Table 1:** Disease association analysis of predicted NKX3.1 targeted genes within the human genome reveals the enrichment of chromosome aberrations, inversion, breakage gene ontology categories

**MeSH Disease/input n = 464**		**Genes**	
	**P-value**	**Expected**	**Observed**
Chromosome inversion	1.67e-04	120	152
Chromosome aberration	2.06e-04	13	27
Angelman Syndrome	2.99e-04	3	10
Chromosome breakage	3.45e-04	20	36
Uniparental disomy	3.95e-04	4	12
Prader-Willi Syndrome	8.64e-04	5	13
Translocation, genetic	9.83e-04	59	82

### Altered expression of predicted downstream target genes in response to NKX3.1 depletion

**Figure 3:**
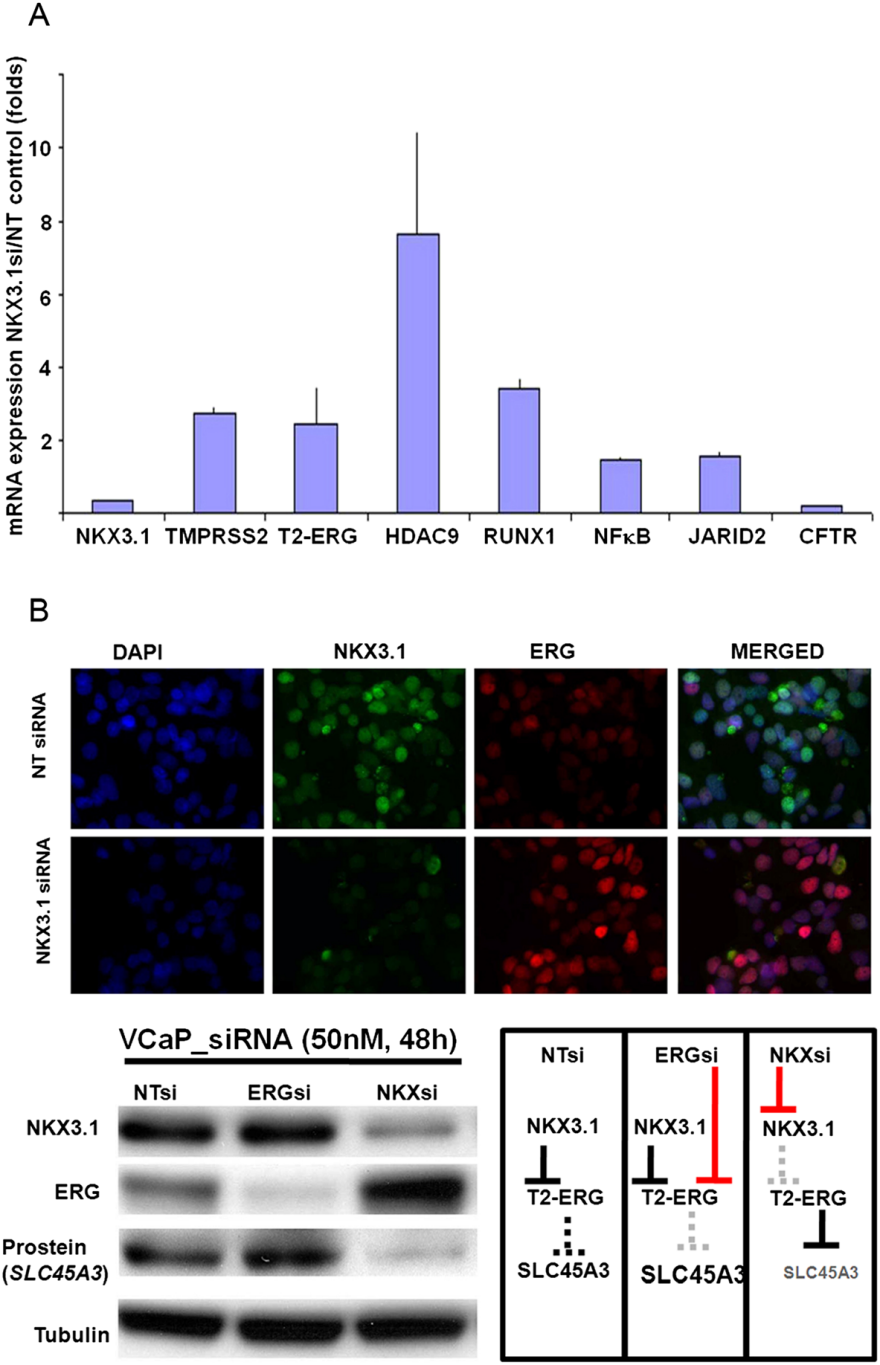
**Expression of predicted NKX3.1 target genes in response to NKX3.1 inhibition. (A)** Depletion of NKX3.1 results in increases in mRNA levels of wild type *TMPRSS2*, *TMPRSS2-ERG* fusion (T2-ERG), *HDAC9*, *RUNX1, NF*к*B* and *JARID.* In contrast, robust reduction of *CFTR* levels is apparent in response to NKX3.1 inhibition. **(B)** Rescue of ERG and its downstream function by NKX3.1 inhibition (NKX3.1 siRNA) is shown by nuclear localization of ERG (upper panel), sharp increases in ERG protein levels (lower panel), and by the depletion of the *ERG*-downstream target prostein (*SLC45A3*). Schematic depiction of the negative regulatory role of NKX3.1 in the context of *TMPRSS2-ERG* (T2-ERG) gene fusion (inset).

To evaluate NKX3.1 in *TMPRSS2-ERG* fusion harboring prostate cancer cells we utilized the siRNA depletion strategy. Consistent with a negative regulatory function of NKX3.1, the transcripts of endogenous *TMPRSS2-ERG* fusion allele, as well as, the wild type *TMPRSS2* showed elevated expression along with *HDAC9*, *RUNX1*, *NF*к*B* and *JARID2* genes in response to *NKX3.1* inhibition (Figure 3A). In line with previous reports we also noted the reduction of *CFTR* expression in response NKX3.1si. This finding suggests that CFTR expression in the human prostate may indeed positively regulated by *NKX3.1*[37]. Gene expression response to NKX3.1 knockdown was noted in approximately half of the examined NKX3.1 target genes. Whole genomic search for model matches in human, rhesus monkey, rat and mouse *TMPRSS2* promoter upstream sequences precisely identified matches of the NKX3.1 model. Thus NKX3.1 as a negative regulator of *TMPRSS2* may evolve in this lineage, since, we found no evidence of model matches within *Tmprss2* promoter upstream regions of zebra fish, opossum, dog and cow genomes. Despite of known informatics constrains, such as, model overfitting and limitations in the employed functional assays the results suggest that comparative analyses for defining conserved repressor elements is a valid approach providing efficient guidance for the experimental validation.

To assess the function of NKX3.1 in regulating the *TMPRSS2-ERG* fusion gene we evaluated ERG expression in response to specific inhibition of NKX3.1. Knockdown NKX3.1 with siRNA resulted in elevated ERG protein levels (Figure 3B). Increased expression and nuclear localization of ERG oncoprotein in response to NKX3.1 siRNA further supported the repressor role of NKX3.1. Consistent with elevated ERG levels we observed marked decreases in prostein. This prostate differentiation associated protein is encoded by the *SLC45A3* gene that is negatively regulated by ERG [22].

### NKX3.1 is a repressor of the *TMPRSS2* gene

**Figure 4:**
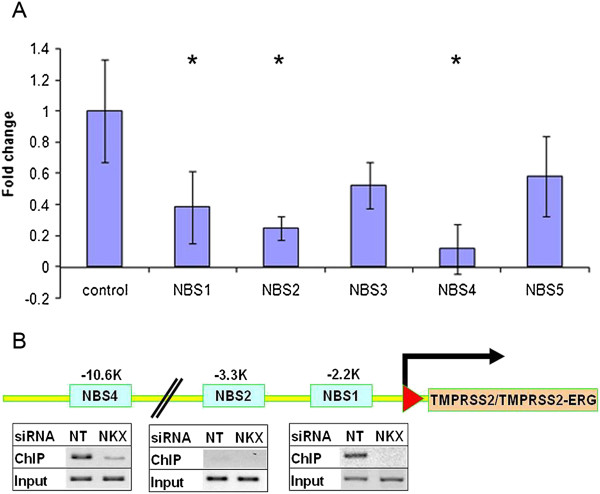
**Predicted NKX3.1 binding sequences of the *TMPRSS2 *promoter are portable repressor elements. (A)** The transcriptional regulatory function of predicted NKX3.1 binding sites (NBS1-5) was assessed by luciferase reporter systems. Relative *luciferase* units are shown as fold changes relative to the control expression levels. Significant (P < 0.05) reduction of reporter gene expression are marked by asterics **(B)** Specific recruitment of endogenous NKX3.1 to predicted NBS1 and NBS4 binding sites of the *TMPRSS2* promoter upstream regions was assessed by *in vivo* ChIP assay in the absence (NT) or presence of NKX3.1 siRNA (NKX).

Although, NBS1 is the only evolutionarily conserved NKX3.1 binding site prediction within the *TMPRSS2* promoter upstream region, transcription factor binding site model match search by MatInspector identified further stand-alone NKX3.1 binding sites. The single matrix prediction identified a tight cluster of five single NKX3.1 matrix model matches (V$NKX31.01) between positions -2298 and -2168 relative to the transcription initiation site that showed partial overlap with NBS1. Further upstream clusters of single NKX3.1 model matches were identified and were designated as NBS2 (-3292; -3277), NBS3 (-8019; -7902), NBS4 (-10684; -10615), and NBS5 (-14628; - 14614). For the assessment of transcription regulatory functions, NBS1-5 sites were cloned upstream to a *Luciferase* reporter vector. The assay result indicated negative regulatory functions for NBS1, NBS2 and NBS4 sequences (Figure 4A). To evaluate the endogenous *TMPRSS2-ERG* gene expression response to NKX3.1 inhibition, VCaP cells were grown in hormone depleted media for three days. Cells were transfected by NKX3.1 siRNA or by non-targeting control siRNA molecules. Synthetic androgen (R1881) was added to the media to induce the expression of androgen regulated genes, including *NKX3.1* and *TMPRSS2-ERG*. After 24 h induction cells were processed for Chromatin Immunoprecipitation (ChIP) assay examining the recruitment of NKX3.1 to NBS1, NBS2 and NBS4. NBS amplicons were excised from the gel and were confirmed by DNA sequencing. The experiment confirmed the recruitment of NKX3.1 to NBS1 and NBS4 regions (Figure 4B).

**Figure 5:**
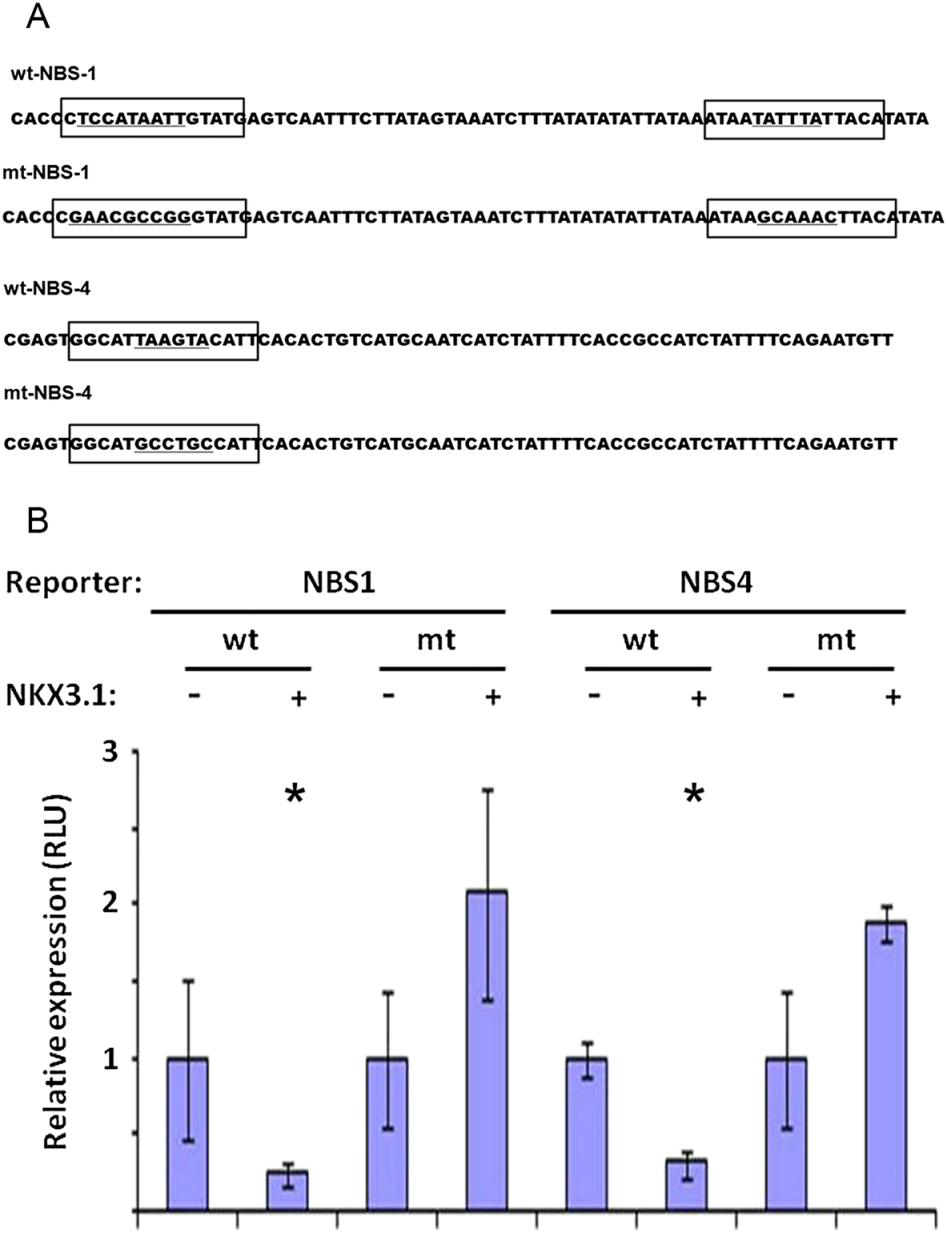
**Both NKX3.1 protein and wild type NKX3.1 binding sites are required for the transcriptional repressor function of *TMPRSS2 *promoter upstream sequences. (A)** Schematic representation of NBS1 and NBS4 sequences marking predicted NKX3.1 binding elements in brackets. Core recognition sequences with transversion mutations are underlined in the wild type (wt) and in the mutant (mt) sequences. **(B)** Relative *luciferase* units (RLU) of wild type and mutant NKX3.1 binding sites were assayed in reporter constructs in the presence (+) of heterologously expressed NKX3.1 or in the presence of control vector (-). Asterisk symbols mark significant (*P* < 0.05) reductions in reporter gene expression.

Although ChIP assays provided an estimated region of recruitment within the chromatin context of NBS1 and NBS4 it does not reveal the actual position and specificity of transcriptional regulatory elements. To address the specificity of NBS1 and NBS4 core binding sites we have introduced transversion point mutations to the core cognate elements aiming to disrupt the NKX3.1 homeodomain DNA recognition (Figure 5A). To reduce the possibility of generating of *de novo* TF binding sites we have used the SeqenceShaper program (http://www.genomatix.de). Wild type and corresponding mutant NBS1 and NBS4 harboring reporter vectors were assayed for reporter gene activity by transfecting HEK293 cells in the presence of NKX3.1 expressing pcDNA-NKX3.1-HA expression vector or control pcDNA. The transfection efficiency was monitored by co-transfecting phRGB-TK *Renilla*-Luc control vector. In the presence of heterologously expressed NKX3.1 the expression of wtNBS1 and wtNBS4 reporters were reduced 4–3 folds, respectively. NBS1- and NBS4-mediated transcriptional repression was disrupted by specific mutations within the V$NKXH core recognition sequences, accompanied by a modest activation in reporter expressions (Figure 5B).

## Discussion

Comparative assessment of evolutionary conserved cognate sequences within the *TMPRSS2* promoter upstream sequences revealed strong conservation of an NKX3.1 binding site. Experimental evaluation of the predicted composite element suggested that this element confers NKX3.1-mediated repression to the *TMPRSS2-ERG* fusion gene in prostate cancer cells. Inhibition of NKX3.1 resulted in elevated expression and nuclear localization of ERG and resulted in reduced levels of the ERG-downstream regulated prostein encoded by the *SLC45A3* gene. Assays for the transcription regulatory function of NKX3.1 binding sites indicated repressor function that was disrupted by specific mutations affecting the DNA recognition of NKX3.1 transcription factor. Recruitment of endogenous NKX3.1 to the evolutionarily conserved cognate element was confirmed by *in vivo* ChIP assay.

**Figure 6:**
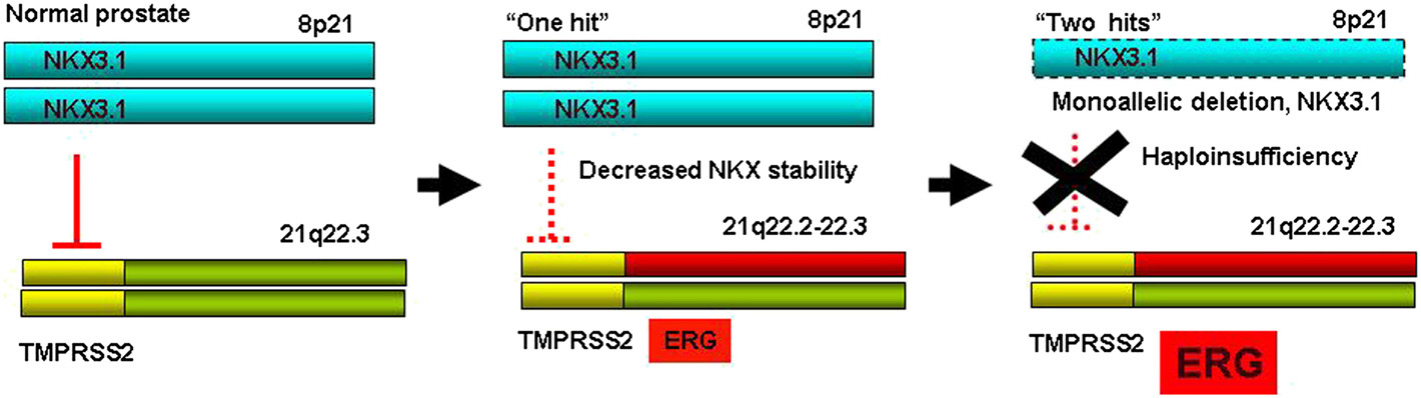
**NKX3.1 haploinsufficiency results in the loss of negative control over the *TMPRSS2-ERG *** **gene fusion.**

Loss of NKX3.1, contributes to the cancer associated function of AR [38,39], C-MYC [21], p53, PTEN [40], Topoisomerase I [41] and TWIST1 [42] in prostate cancer. *ERG* oncogene, a result of the *TMPRSS2-ERG* fusions, negatively regulates *NKX3.1* through EZH2 [25]. In the current study we have examined evolutionary conserved composite regulatory models of the *TMPRSS2* gene. The analysis revealed a remarkable conservation of a composite model with an NKX3.1 binding site in the lineage of mouse, rat, rhesus monkey and human species members of the Euarchontoglires (Supraprimates) *super ordo*. This composite model identified sequences within intronic regions of the human genome. Increased expression of evaluated NKX3.1 target genes (*HDAC9*, *RUNX1*, *TMPRSS2*, *TMPRSS2-ERG*, *NF*к*B* and *JARID2*) was observed in response to NKX3.1 inhibition. Meta-analysis of Nkx3.1 target genes from *in vivo* ChIP assay of mouse prostates indicated that upstream regulatory regions are indeed enriched in core elements, such as, V$NKXH, V$HOXF and V$BRNF (Table S3 in [21]) similar to the model we have obtained from *in silico* analysis. Pathway analysis of NKX3.1 target genes from the current study, as well as, from the reported *in vivo* model [21] revealed NFкB as the central regulatory node of NKX3.1 target gene signatures. Furthermore, the analyses indicated, robust enrichment of genes controlling chromosomal integrity. These findings are consistent with the reported role of NKX3.1 in cellular response to DNA damage [20,41]. These observations are also consistent with an NFкB-mediated protective function of NKX3.1 linked to inflammation and tumorigenesis [15,43-47]. Taken together our study highlights NKX3.1 as a negative regulator of the*TMPRSS2* promoter. Thus, the frequently observed haploinsufficiency of *NKX3.1* in prostate cancer may significantly contribute to the activation of *ERG* protooncogene in the *TMPRSS2-ERG* fusion genomic context. This finding highlights the integrated role of *TMPRSS2-ERG* gain and *NKX3.1* losses as cooperating events in prostate tumorigenesis (Figure 6).

## Conclusions

Approximately half of the prostate cancer cases harbor the *TMPRSS2-ERG* gene fusions in Western countries. This recurrent oncogenic event leads to the activation of the *ERG* oncogene. In the current study evaluation of conserved regulatory elements of *TMPRSS2* promoter upstream sequences revealed conservation of binding sites for the NKX3.1 tumor suppressor. NKX3.1 binds to these sequences and represses the *TMPRSS2-ERG* fusion gene. Thus, the frequently observed loss of NKX3.1 in prostate cancer may significantly contribute to the activation of *ERG* protooncogene. Pathway analysis of NKX3.1 target genes from the current study, as well as, from the reported *in vivo* studies revealed NFкB as the central regulatory node of NKX3.1 target gene signatures with robust enrichment in genes controlling chromosomal integrity. These findings suggest that *TMPRSS2-ERG* gain and NKX3.1 losses are potentially cooperating genetic events in prostate tumorigenesis.

## Methods

### Cell lines, cell culture and reagents

Human prostate tumor cell line, VCaP and human embryonic kidney HEK293 cells were obtained from the American Type Culture Collection (ATCC, Rockville, MD) and were maintained in growth medium and under conditions recommended by the supplier. The synthetic analogue of androgen, R1881, was purchased from New England Nuclear (Boston, MA).

### Inhibition of NKX3.1 and ERG with small interfering RNA and heterologous expression of *NKX3.1*

Small interfering RNA (siRNA) oligo duplexes against human *NKX3.1*(L-015422-00), and Non-targeting control siRNA (D-001206-13-20) were from Dharmacon (Lafayette, CO), ERGsi RNA as previously described [22]. Transfection or co-transfection of 50 nM siRNAs and 1 μM of plasmids was carried out with Lipofectamine 2000 (Invitrogen, Carlsbad, CA) in triplicates. The wild type human *NKX3.1* expressing vector pcDNA3.1-NKX3.1-HA was a kind gift from Dr. Charles J. Bieberich, University of Maryland Baltimore County, Baltimore, Maryland. In six-well plates HEK293 cells were transfected in triplicates with the pcDNA3.1 control or with the pcDNA3.1-NKX3.1-HA expression vectors by using Lipofectamine 2000. Cells were harvested for protein and mRNA analysis after 48 h incubation.

### Chromatin immunoprecipitation assay

For assessing the specific recruitment of endogenous NKX3.1 to the predicted NKX3.1 binding sites *in vivo* ChIP assays were carried out in the presence of NKX3.1 siRNA or control NT siRNA [35]. VCaP cells were grown in 10% charcoal stripped serum (cFBS) containing media (Gemini Bio-Products, Carlsbad, CA) for 48 h and were transfected with 50 nM NKX3.1 siRNA or 50 nM of NT control. Cells were incubated for 24 h followed by the addition of 0.1 nM of R1881. At the 48 h time point following hormone induction formaldehyde was added to the cell culture media to 1% and the cells were processed for ChIP assay [48] by using the mouse monoclonal anti-ERG antibody (CPDR ERG-MAb, clone 9FY, currently available from Biocare Medical, Concord, CA) [7]. NBS1 region from input and ChIP DNA samples were amplified by the forward 5’-TGTTTCTCTGGAGAACCCTGA-3’ and reverse 5’- GCAGGTGCAGTTGTCTTTCA-3’; NBS2 region was amplified by the forward 5’- CAATCCAGGCAGGGCTATTA and reverse 5’- GGGCAATAGCTGGTGTTTGT-3’; the NBS4 region was amplified by the 5’- TCATCTATTTTCACCGCCATC-3’ and 5’- ACACGCACACACCACATCAT-3’ primer pairs under previously described PCR conditions [22,35].

### Assessment of the transcription initiation site of *TMPRSS2-ERG* transcript by 5’ oligocapping

Under approved protocol from the WRAMC IRB six cases were identified with *TMPRSS2-ERG* fusion harboring prostate tumors. Total RNA was isolated from the tumors and were pooled [29]. From the pool 4.2 μg of total mRNA was subjected to 5’ oligocapping procedure (FirstChoice, RLM-RACE, Ambion, Austin, TX) pairing the 5’-GGCGTTGTAGCTGGGGGTGAG-3’ [11] with the outer, and 5’- CAATGAATTCGTCTGTACTCCATAGCGTAGGA-3’ with the inner primer. Amplicons were gelpurified and cloned into pUC19 vector and were subjected to DNA sequencing in forward and reverse directions.

### Comparative analysis of the *TMPRSS2* gene promoter upstream sequences

DNA sequences of the 15,000; +78 bp region of *Homo sapiens*, *Macaca mulatta*, *Rattus norvegicus* and *Mus musculus* genomes were extracted from the NCBI build 36.3 database. Scanning from the proximal promoter towards the distal sequences 3,000 bp homologue segments were evaluated allowing 500 bp overlap of segments at each composite model scanning step. DNA sequence segments of all examined species were analyzed by the FrameWorker (version 5.4.3.3, http://www.genomatix.de) for conserved composite model matches by using the Matrix Family Library 7.1 at the following settings: core promoter elements 0.75/optimized, vertebrates (0.75/optimized); distance between adjacent elements: 5–200; distance band with: 10, exhaustive model search with minimum number of elements = 2 and max number of elements = 6. Overall the highest number of common single element match was the V$NKXH, a binding site for NKX3.1. Ranking the composite models revealed only one model that reached the maximum (four element) complexity. The top scoring model was defined as V$HOXF (strand orientation (+), distance to next element 43-51 bp), V$NKXH (strand orientation (-), distance to next element 7-14 bp), V$PARF (strand orientation (-), distance to next element 17-23 bp); V$BRNF (strand orientation (-), distance to next element 0 bp) at settings of minimum core similarity = 0.75 and minimum matrix similarity “optimized”. Next the entire human genome (NCBI build 36.3) was searched with this composite model for matches by the ModelInspector 5.6 program (http://www.genomatix.de). Whole –genomes model searches confirmed the model match within the *TMPRSS2* gene promoter upstream sequences in *Homo sapiens*, *Macaca mulatta*, *Rattus norvegicus* and *Mus musculus* genomes and indicated the absence of model match within the *Tmprss2* gene loci of *Canis lupus familiaris*, *Bos Taurus*, *Monodelphis domestica*, and *Danio rerio*.

### Pathway and meta-analyses of NKX3.1 genomic targets

Predicted gene targets for NKX3.1 were obtained by *in silico* composite model match analysis of the entire human genome. Among the total 1636 (1371 non-redundant) model matches 559 were non-annotated. Within the annotated 1037 model matches (Additional file 2: Table S1) 627 was found in intronic, 10 and 12 matches were found in exonic or promoter sequences, respectively. Intronic, exonic and promoter model matches were further filtered for genes with defined gene symbols and the final set of 452 genes were used as input for pathway analysis (Additional file 2: Table S2). Prostate Cancer meta-analysis dataset used in our study was based on the report of Anderson et al. [21]. NKX3.1 target genes were imported into the Genomatix Pathway System (GePS, http://www.genomatix.de). In GePS genes were mapped into networks based on the information extracted from public databases including National Cancer Institute Pathway Interaction Database (http://pid.nci.nih.gov) and Biocarta (http://www.biocarta.com). The generated network displayed as nodes and connections focused on functional relationships between genes based on the number of evidences in literature (Figures S1 and S2). For the analyses we have used function word evidence level to generate the network where gene pairs are noted if they occur in the same sentence connected with a function word.

### Immunoblot assay

At the specified time points VCaP cells treated with NKX3.1si or control NTsi were lysed in M-PER Mammalian Protein Extraction Reagent (Pierce, Rockford, IL) supplemented with protease (Roche Applied Science, Indianapolis, IN) and phosphatase inhibitor cocktails (Sigma, St. Louis, MO). ERG proteins were detected by Western blot (NuPAGE Bis-Tris gel, Invitrogen) as described previously using immunoaffinity-purified anti- ERG mouse monoclonal antibody 9FY [7]. The anti-NKX3.1 polyclonal antibody (T-19) and anti-alpha tubulin (B-7) antibodies were obtained from Santa Cruz (Santa Cruz, CA) and the anti-prostein antibody recognizing the protein product of the *SLC45A3* gene was obtained from DAKO (Carpinteria, CA). Representative images of two independent experiments are shown in the Results.

### Immunofluorescence assay of siRNA treated VCaP cells

VCaP cells were fixed in 4% paraformaldehyde and centrifuged onto silanized slides (Sigma, St.Louis, MO) with a cytospin centrifuge. Cells were immunostained with anti-ERG (9FY) and anti-NKX3.1 (Santa Cruz) followed by goat anti-mouse Alexa-488 and anti-goat Alexa-594 secondary antibodies (Invitrogen, Carlsbad, CA). Images were captured by using a 40X/0.65 N-Plan objective on a Leica DMLB upright microscope with a QImaging Retiga-EX CCD camera (Burnaby, BC, Canada) controlled by OpenLab software (Improvision, Lexington, MA). Images were converted into color and merged by using Adobe Photoshop.

### NKX3.1 binding site (NBS) *luciferase* reporters and dual-*luciferase* reporter assays

Mutant NBS sequences were designed to minimize the generation of artificial binding sites by the Sequence Shaper (http://www.genomatix.de). Wild type and mutant NBS sequences were chemically synthesized adding a cohesive overhang for *Nhe1* site (CGCGT) at the 5'-end of the sense strand and an overhanging Bgl2 site (TCGAG) at the 3‘ as follows: wild type NBS1 5’-CTCCATAATTGTATGAGTCAATTTCTTATAGTAAATCTTTATATATATTATAAATAATATTTATTACATATAAGCTGTGTATAATATATATCAT-3’; mutant NBS1 5’-**GAACGCCGGG**TATGAGTCAATTTCTTATAGTAAATCTTTATATATATTATAAATAA**GCAAA**CTTACATATAAGCTGTGTATAATATATATCAT-3’ ; wild type NBS2 5’-CACATAACTTAAGGCATATTGACTTTATATCATTGTATTAAGTATTGTTAATTTTACATTA-3’; mutant NBS2 5’-CACATAAAGGCCTGCATATTGACTTTATATCATTGGCGGCCTTATTTGGCCGGTTACATTA-3’; wild type NBS3 5’-CGAGAAAAGGATTCAAATACTTAGGAAGATTGAAATGTGAGGGT-3’; mutant NBS3 5’-CGAGAAAAGGATTCAAA**GCCGGC**GGAAGATTGAAATGTGAGGGT-3’; wild type NBS4 5’- CGAGTGGCATTAAGTACATTCACACTGTCATGCAATCATCTATTTTCACCGCCATCTATTTTCAGAATGTTCTCA-3’; mutant NBS4 5’- CGAGTGGCAT**GCCTGC**CATTCACACTGTCATGCAATCATCTATTTTCACCGCCATCTATTTTCAGAATGTTCTCA-3’; wild type NBS5 5’-CAAAACCAAATACTGCATGTTCTCACTTATAAGTGGGAGCTGGACAATGAGAACACATGGACACAGGGAGA-3’; mutant NBS5 5’-CAAAACCAAATACTGCATGTTCTAACAGGCTACTGTGGAGCTGGACAATGAGAACACATGGACACAGGGAGA-3’. The 5’ end of synthetic oligonucleotides were phosphorylated by using polynucleotide kinase, the complementary strands were annealed and gelpurified and ligated to the NheI-BglII sites of the gelpurified, phRG-TK reporter (Promega, Madison, WI). The phRG-TK vector is a synthetic reporter vector that has been designed to minimize binding sites for transcription factors. HEK293 cells were transfected with the reporter and pGL3 luciferase control vectors in triplicates. Forty-eight hours after the transfection, the activities of control phRG-TK reporter *Renilla luciferase* and pGL3 *Firefly luciferase* constructs were determined by the Dual-Luciferase Reporter Assay system (Promega, Madison, WI). Cells were rinsed with phosphate-buffered saline, and lysed with 1 × passive lysis buffer. Twenty μl of cell lysates were transferred into the luminometer tube containing 100 μl luciferase assay reagent II. Firefly luciferase activity (N1) and were measured first, and then *Renilla luciferase* activities (N2) were determined after the addition of 100 μl Stop & Glo reagent. N2/N1 light units were averaged from three measurements and were expressed as relative luciferase units (RLU).

### RNA extraction, reverse transcription and real-time PCR quantification

Total RNA was extracted from cell monolayer using Trizol® total RNA isolation reagent (Gibco BRL, Life Technologies, Gaithersburg, MD, USA) as per the manufacturer's protocol. Real-time PCR was performed in triplicates using an Applied Biosystems 7300 Sequence Detection system using SYBR green PCR mix (Qiagen) or by TaqMan assay (Applied Biosystems). The expression of *GAPDH* was simultaneously analyzed as endogenous control, and the target gene expression in each sample was normalized to *GAPDH*[49]. RNA samples without reverse transcription were included as the negative control in each assay. Amplification plots were evaluated and threshold cycle (CT) was set for each experiment. Measurements for target gene and *GAPDH* endogenous control were averaged across triplicates and standard deviation for each set was calculated. ΔCT values were calculated by subtracting averaged *GAPDH* CT from averaged target gene CT and expression fold-change differences were calculated by comparing ΔCT values among sample sets. Primer pairs for the amplification of target genes were as follows. *HDAC9*: forward 5’- CAAATGGTTTCACAGCAACG -3’, reverse 5’- TGCGTCTCACACTTCTGCTT -3’; *JARID2*: forward 5’- AGGAGACTGGAAGAGGCACA -3’ and reverse 5’- GTCCGTTCAGCAGACCTCTC -3’; *NF*к*B*: forward 5’- TATGTGGGACCAGCAAAGGT -3’ and reverse 5’- AAGTATACCCAGGTTTGCGAAG -3’; *RUNX1* forward 5’- CAGATGGCACTCTGGTCACT-3’ and reverse 5’- TGGTCAGAGTGAAGCTTTTCC-3’; *CFTR* forward 5’- CCAGATTCTGAGCAGGGAGA-3’; reverse 5’- TTTCGTGTGGATGCTGTTGT-3’. Primers and probes for *TMPRSS2* and *TMPRSS2-ERG*, as well as for NKX3.1 have been described before [50,51].

### Statistical analysis

Gene expression analyses results are shown by bars representing mean+/- S.E., from three independent experiments (n = 3). Anova and Dunnett t test were applied for statistic analysis using the SAS software (http://www.sas.com). Significant gene expression differences, *P* < 0.05, are marked with asterisk. Enrichment scores and *P*-values of the bioinformatics analyses were calculated by the Genomatix Software (http://www.genomatix.de).

## Abbreviations

NKX3.1: NK3 Homeobox 1; Transmembrane protease: serine 2; NBS: NKX3,1 binding site; ERG: V-Ets Erythroblastosis Virus E26 Oncogene Homolog (Avian); SLC45A3: Solute carrier family 45, member 3; JARID2: Jumonji, AT Rich Interactive Domain 2; NFкB: Nuclear Factor of Kappa Light Polypeptide Gene Enhancer In B-Cells 1; HDAC9: Histone Deacetylase 9; RUNX1: Runt-Related Transcription Factor 1; CFTR: Cystic Fibrosis Transmembrane Conductance Regulator (ATP-Binding CassetteSub-Family C, Member 7); TSR: Transcriptional start region; TSS: Transcriptional start site; HEK293: Human Embryonic Kidney 293 cell line; VCaP: Vertebral-Cancer of the Prostate cell line.

## Competing interest

AD, S-HT and SS are coinventors of the ERG-MAb 9FY, licensed by the Biocare Medical Inc.

## Authors’ contributions

AD and SS designed research. RT, FS, GP and AAM performed experiments. S-HT contributed with the new ERG-MAb reagent and critical experimental procedures. RT, SK and AD performed bioinformatics experiments. RT and AD analyzed data. AD and SS wrote the paper. All authors read and approved the final manuscript.

## Pre-publication history

The pre-publication history for this paper can be accessed here:

http://www.biomedcentral.com/1471-2407/14/16/prepub

## Supplementary Material

Additional file 1: Figure S1 NFкB forms the central node of predicted NKX3.1 target genes within the human genome. Click here for file

Additional file 2: Table S1 IDs of annotated genes (1037) obtained from the list of non-redundant model matches of predicted NKX3.1 targets within the human genome. The TMPRSS2 gene ID is underlined on chromosome 21. Click here for file
