# Supplementary material for: Loss of the NKX3.1 tumorsuppressor promotes the TMPRSS2-ERG fusion gene expression in prostate cancer
Source: BMC Cancer. 2014 Jan 13;14:16. doi: 10.1186/1471-2407-14-16 (PMC3897978; doi:10.1186/1471-2407-14-16)

Additional files

Additional file 1 as PDF

Additional file 1: Figure S1 NFκB forms the central node of predicted NKX3.1 target genes within the human genome.

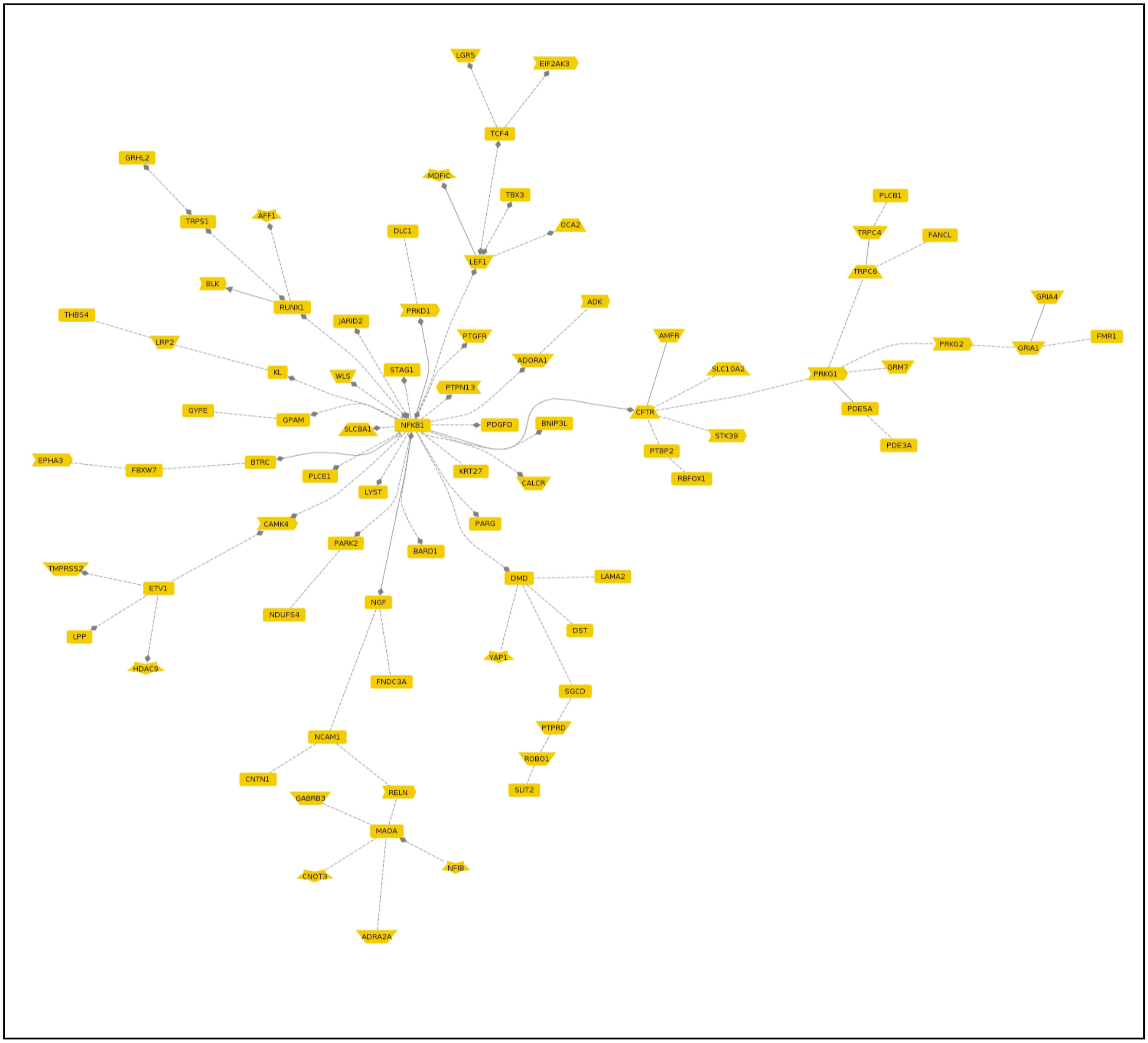

Additional file 1: Figure S2. Nfκb forms the central node of Nkx3.1 target gene signature obtained by ChIP-Seq analysis of Nkx3.1 recruitment to the mouse genome (Anderson PD, *et al.* (2012) Nkx3.1 and Myc crossregulate shared target genes in mouse and human prostate tumorigenesis. *The Journal of clinical investigation* 122(5):1907-1919).

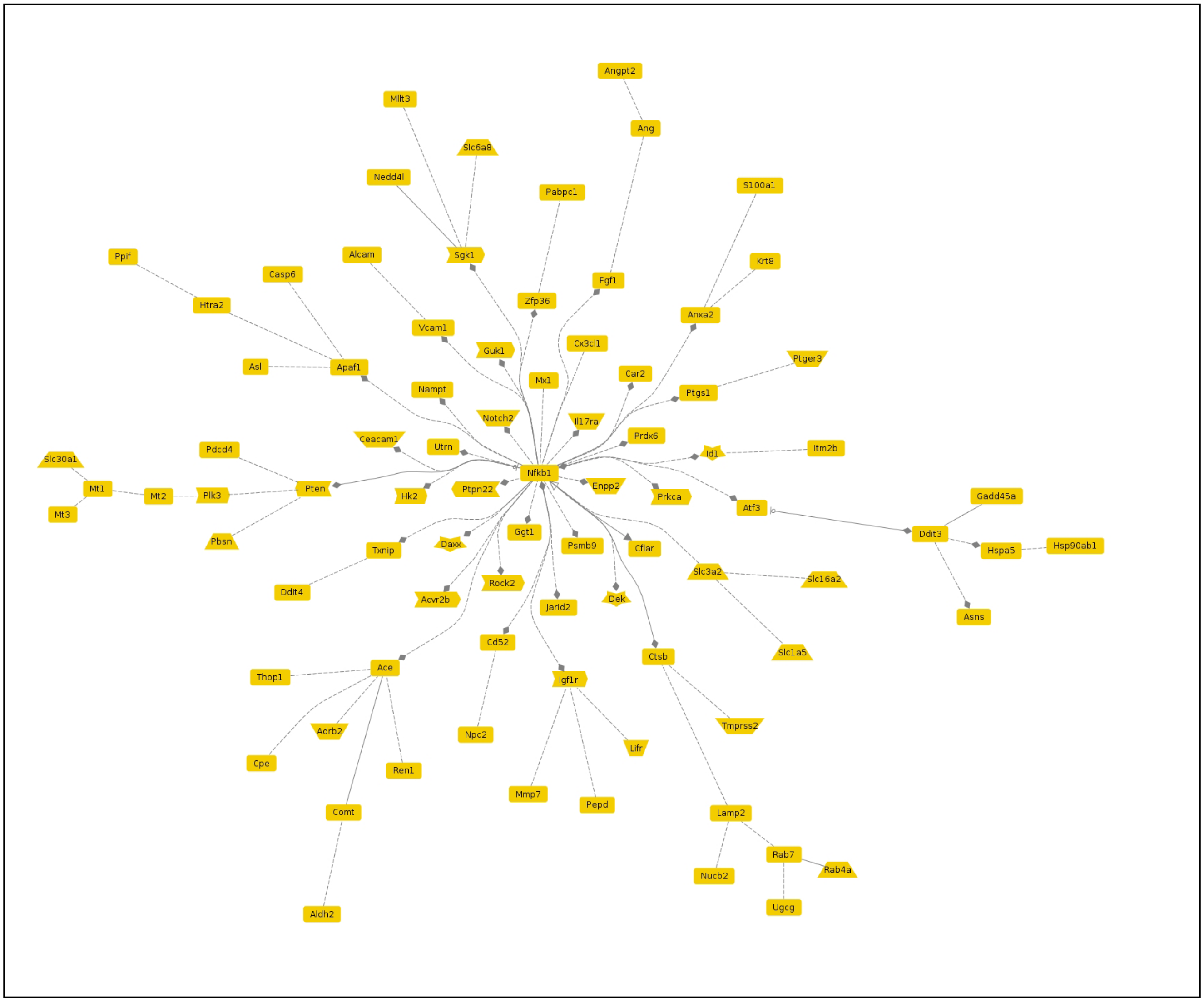

Supplement: Additional file 1: Figure S1 — NFкB forms the central node of predicted NKX3.1 target genes within the human genome. [file 1471-2407-14-16-S1.pdf]
