# Supplementary material for: Loss of the NKX3.1 tumorsuppressor promotes the TMPRSS2-ERG fusion gene expression in prostate cancer
Source: BMC Cancer. 2014 Jan 13;14:16. doi: 10.1186/1471-2407-14-16 (PMC3897978; doi:10.1186/1471-2407-14-16)
Supplement: Additional file 2: Table S1 — IDs of annotated genes (1037) obtained from the list of non-redundant model matches of predicted NKX3.1 targets within the human genome. The TMPRSS2 gene ID is underlined on chromosome 21. [file 1471-2407-14-16-S2.pdf]

Additional file 2 as PDF

Additional file 2 Table S1. IDs of annotated genes (1037) obtained from the list of non-redundant model matches of predicted NKX3.1 targets within the human genome. The *TMPRSS2* gene ID is underlined on chromosome 21.

Chromosome 1:
1441, 2899, 100133966, 400750, 10450, 728621, 84871, 11124, 4898, 5865, 51060, 79699, 6342, 51668, 23358, 8613, 1600, 205,729620, 79971, 57554,257194, 642098, 127255, 127253, 431707, 204962, 26289, 26009, 5737, 64123, 729779, 23266, 79739, 80135, 255631, 676, 7813, 58155, 126987, 55170, 4803, 6699, 6703, 83540, 729952, 22920, 51430, 9910, 57795, 400796, 85397, 1660, 116496, 5321, 339479, 51022, 79577, 343450, 54530, 400800, 23271, 134, 26254, 55733, 1063,55105, 5867,375061,440730, 57568, 1130,81469,441933

Chromosome 2:
54221, 23040, 400941, 129607, 400945, 23369, 151325, 51232, 100131085, 64225, 8491, 100128590, 6546, 27436, 11036, 9378, 98,57223, 114800,7444,55120, 84141, 6936, 80059, 1496, 388965, 51652,100134301, 440871, 9451, 50802,650405, 100131408, 442028, 3899, 8809, 60482, 442038,402100, 84220, 729540,652919, 727747, 57628, 8886,728241, 129684, 2995, 100134031, 554226, 344148, 81615, 130013, 84083, 647012, 53353, 8942, 728773, 100133235, 391453, 56475, 114805, 151531, 55137, 2888, 6332, 643496, 27347, 4036, 29789, 220988, 91752, 285135, 401024, 55854, 165215,66037, 5334,4999,79800, 117583,8828,22868, 130749, 580,26154, 6508, 2043, 653773, 2181, 80309,55022, 100128709, 401037, 51281

Chromosome 3:
27255, 152330, 9695, 2917, 401050, 9901, 100134121, 151651, 151648, 391518, 7325, 27303, 7048, 339896, 201595, 54918, 25827, 23122, 10015, 10777, 3680, 200844, 23024, 5067, 401076, 6092, 6091, 2632, 253559, 2042, 63899, 389137, 644063, 391556, 285220, 84319, 11259, 644444, 25890, 131368, 214, 9666, 401082, 389141, 90102, 285335, 728873, 152404, 9515, 6565,286676, 84303,60678, 256076,348808, 66000, 10274,646547,51163, 1360,116931, 170506, 344758, 4311,389168,730086, 389170, 151742, 6476, 22865, 93556, 2122, 22871,647212, 254827, 730168, 131118, 64108, 6750,4026, 2257, 151963,57110, 131583, 152002

Chromosome 4:
644753, 391634, 952, 9982, 729006, 5860, 254251, 645174, 9353, 80333, 55296, 401123, 5099, 645716, 642305, 727819, 401124, 57495, 51088, 132789,2565, 10699,255130,23284, 644578,65008,80144,255119, 5593,5783,4299, 10144, 401145, 2895, 658, 4790, 1062, 6870, 51176, 3426,64579, 133022, 9348, 100131243, 57721, 2169, 8654,4085, 100128580, 166378,645762,57182, 646187, 57575, 729578, 54510, 23657, 3600, 8821, 2996, 285423, 166614, 55294, 6423,54798,5356, 2982, 2983, 1519, 646865, 9693, 56884, 55016, 50859, 91351, 23022, 51166, 442117, 80817, 3248, 11086, 2823, 60559, 7424, 285501, 728081, 728191, 55714, 4543

Chromosome 5:
79192, 340094, 729506, 100128382, 50834, 134145, 1611, 1501, 10409, 285697, 391769, 1016, 728411, 1010, 391771,439936,100133463, 100131678, 1007, 729862, 100130803, 79925, 729,345557, 10884,348980, 133418,3670,3672,4724, 153129, 115827, 5144,79993,91942, 10283, 373,375449, 100129571, 653238, 170591, 8546,9456,

100129870, 7060, 23635,645079, 731042, 4208, 729011,441097, 83594, 9366, 91137, 85480, 814,324,3781,57556,728342, 340069, 51334, 57507, 65983, 2201, 28965, 337876, 3094, 56132, 56131, 9832, 2890, 9421, 23367, 6444, 285626, 147,27430, 728843, 6586, 54908, 30820, 2568, 64901, 153579

Chromosome 6:

8899, 285780, 389365, 266553, 2651, 3720, 54901, 389370, 4276, 10866, 7629, 11329, 114781, 2739, 442213, 245915, 83741, 7021, 646517, 55227, 653, 81578, 667, 221336, 727842, 727798, 202559,100128610, 642443, 100128757, 728052, 55788, 135228, 1303, 3617,643281,3351, 134728, 100134204, 100131508, 728360, 9892, 22832, 9096,100127905, 643926, 6885,2045,79694,442236, 253714, 389419, 57531, 5550, 639, 85413, 442249, 728590, 221322, 5570, 154215, 3908, 93663, 2070, 6943, 167826, 100130476, 167838, 10370, 729070, 729076, 7402, 7957, 23345, 80177, 26575, 389435, 4988,51106,5340,4216,5071, 10846, 90632, 6196, 64094, 100132959

Chromosome 7:

260341, 401296, 30010, 168741, 340268, 100134489, 221981, 54664, 2115, 57037, 9734, 222894, 221830, 56164,4852, 11112, 9805, 64224, 989, 11281, 8621, 5478, 94239,285877, 100129159, 100134659, 728416, 100133482, 100128613, 27320, 100134664, 442319, 7697,100134576, 26053, 9569, 5380, 641808, 9863, 10512, 100134557, 223117, 2913, 9988, 799, 10282, 1278,57001, 6863, 222234, 5649, 375612, 29969, 1080, 83992, 51691, 56311, 3751, 50833, 6561, 64753, 54927, 56829,57180,346547,140545

Chromosome 8:

640, 10395, 23362, 4023, 6570, 100132107, 10863, 665, 79618, 642513, 100127894, 137107, 100133273, 203100, 56892, 79698, 54212, 137902, 9705, 4986,100129667, 157708,6101, 157503, 137695, 4067, 6224, 90362, 286187, 79848, 27067, 54968, 83690, 3174,79776,5569,51101, 138046, 441363, 85481, 8895, 168975, 4325,8767,441369, 79815, 3788,79977, 83988, 7201,84955, 93035, 27012, 114788, 728795, 7227, 8667, 401474, 441377, 4982, 10584, 594842, 100131552, 340359, 312,157638, 5462,728724, 100129525, 56169

Chromosome 9:

9933, 5991, 26953, 90865, 90871, 5789, 4781, 158326, 729983, 100131640, 1993, 100129669, 79817, 158038, 401497, 5646,392309, 100130309, 100132851, 727745, 26149, 653114, 100133160,389741, 100132072, 642929, 100133021,441420, 100133312, 643630,728903, 100131538, 643792, 100131760, 442421, 392335, 548321, 84210, 220869, 23670, 645345, 7091, 347119, 7088, 55582, 60560,53358, 1164, 100132701, 4920, 401541, 11046,54886, 1539, 10592, 1620,55755,392392, 5082, 5090, 89853

Chromosome 10:

727894, 728544, 389936, 644540, 9712, 83641, 84898, 57512, 91452, 220929, 8453, 8325, 91074, 441666, 100133130, 653129,728813, 338598, 100133189, 100134210, 55747, 653365, 728725, 399761, 644021, 399753, 55753, 8505, 653252, 653308, 29974, 5592, 65217, 11130,253430, 22891, 29119,56521,219690,219699, 132, 10718, 728027, 54462, 2894, 23063, 355, 9023,51196, 404033, 100127889, 8945, 79591, 22986, 114815, 150, 57678, 4892, 57700, 26033, 22841, 256536

Chromosome 11:

645581, 338645, 63982, 159963, 387758, 627, 81930, 54765, 100129825, 100131023, 57689, 387761, 51144, 374393, 63901, 2352, 2348, 10825, 100129795, 7405, 51773, 26011,

220382, 8301, 143686, 143684, 8690, 53942, 7225,10413,79659,80310,2893, 100049615, 399947, 4684, 6653, 399959, 390259, 3312,387820,2113,729305

Chromosome 12:
100129645, 894, 57103, 10635, 118426, 50865, 93164, 83445, 2012, 51071, 5139, 3709, 636, 121512,144402, 1272, 196528, 51474, 51411, 4327, 84305, 91419, 121227, 9194,338811, 23426, 55832, 56890, 5801, 8549, 29953, 100130268, 114882, 144455, 89795, 160335,55117, 160418, 4254,728084, 1848,643153, 439916, 694, 574016, 574028, 338809,732096, 121456, 56899, 121601, 6926, 100129020, 84530, 160777, 387890, 2054,5901

Chromosome 13:
284232, 348021, 55269, 9365, 54937, 29880, 7223, 387921, 10186, 80183, 647262, 22862, 81617, 2974, 115825, 144983, 10562, 100128485, 122183,341689, 100134278, 81550, 144766, 5101,400141,57626, 10464, 4008,64062,10253,647313, 114798, 387939, 84189, 100133947, 144776, 2262, 10082,55757,266722, 84899, 390423, 6555, 728183, 267012, 23026, 8660

Chromosome 14:
440153, 387978, 5587, 9472, 64067, 161198, 283547, 145581, 645086, 23116, 732210, 651787, 161357, 6235,9147, 122786, 54331, 57161, 54916, 9786, 51339, 440181, 23002, 4331, 81537, 83544, 9517, 9369,1734, 145508, 730105,23768,55727,767564,767565,1735, 145200,5891, 100131845,90925

Chromosome 15:
339010, 6638, 2562, 2567, 4948, 54893, 1139, 57099, 1133, 89978, 4212, 825, 6557, 100130218, 2200, 9728, 9318, 645693, 256764, 64864, 8925, 26035, 123722, 123624, 145978,4916,8826,64784

Chromosome 16:
54715, 10368, 5930, 27327, 9951, 162083, 6817, 728734, 400509, 64755, 51327, 386757, 647591, 91807, 388272, 29117, 79068, 267,5432,390735,729217,54768,388289,441506, 10428, 57687

Chromosome 17:
4991, 8390, 6368, 342574, 147184, 3859, 8405, 339209, 645173, 64122

Chromosome 18:
56651, 64863, 5797, 284222, 56907, 388468, 400858, 729863, 644669, 400645, 1823, 374864, 56853, 647946, 641516, 6860, 26040, 8170,220134, 5596, 100130003, 284254, 6925, 100134353, 9352, 5273,221241, 1005, 28513, 92126, 54495, 79839, 220164, 220158, 147381,79863

Chromosome 19:
30817, 199777, 163223, 100131141, 727780, 7386, 284395, 9745, 57616, 22900, 163071, 284370, 4849

Chromosome 20:
650, 54363, 56255, 23236, 728434, 728573, 5126, 57186, 55857, 284801, 400841, 140823, 9584, 60625, 9935, 149699, 84969, 10955, 79716, 149775, 128611

Chromosome 21:

149992, 729501, 642460, 317754, 4685, 400860, 861, 6450, **7113(TMPRSS2)**

Chromosome 22:
81061, 644768, 26080, 642633

Chromosome X:
412, 26609, 4281, 395, 6322, 10742, 6197, 22866, 139420, 439944, 11141, 1756, 645090, 442444, 392447, 286444, 8573, 80316, 4128,79742,392452, 158586,7789,55613,55906, 53344,646193, 546, 10800,203430, 6451,5456,53336,90316,100130134, 140886,392501, 401602, 100130919, 649076, 100130176, 9643, 26280, 84187, 392522, 55849, 154796, 286528, 11254, 653155, 90293, 65109, 7737, 2747,2892, 10178, 100129520, 340578, 100130613, 139741, 399668, 203522, 9459, 286411,51438,441525,494118, 84631, 2332, 158521, 2334, 8776, 83692, 1193,7411

Chromosome Y:
438, 392501, 100101121, 8653, 7404, 22829, 140032, 159125

Additional file 2 Table S2. Input gene IDs (1-452) with gene symbols used in the GePS pathway analysis (www.genomatix.de) representing intronic, exonic and promoter matches for the NKX3.1 binding site model within the human genome.

#	Gene ID	Gene Symbol
1	98	ACYP2
2	132	ADK
3	134	ADORA1
4	150	ADRA2A
5	205	AK4
6	267	AMFR
7	324	GXL 1234159
8	373	TRIM23
9	395	ARHGAP6
10	438	ASMT
11	580	BARD1
12	640	BLK
13	658	BMPR1B
14	665	BNIP3L
15	667	DST
16	676	BRDT
17	799	CALCR
18	814	CAMK4
19	825	CAPN3/GANC
20	861	RUNX1
21	1010	CDH12
22	1063	CENPF
23	1080	CFTR
24	1130	LYST
25	1133	CHRM5
26	1139	CHRNA7
27	1193	CLIC2
28	1272	CNTN1
29	1303	COL12A1
30	1360	CPB1
31	1600	DAB1/OMA1
32	1756	DMD
33	1823	DSC1
34	2042	EPHA3
35	2070	EYA4
36	2115	ETV1
37	2169	FABP2
38	2257	FGF12
39	2332	FMR1
40	2562	GABRB3
41	2567	GABRG3
42	2651	GCNT2
43	2890	GRIA1
44	2893	GRIA4
45	2894	GRID1
46	2895	GRID2
47	2917	GRM7
48	2974	GUCY1B2
49	2996	GYPE
50	3426	CFI
51	3617	IMPG1
52	3680	ITGA9
53	3709	ITPR2
54	3720	JARID2
55	3751	KCND2
56	3899	AFF3
57	3908	LAMA2
58	4008	LMO7/UCHL3
59	4026	LPP
60	4036	LRP2
61	4128	MAOA
62	4212	MEIS2
63	4281	MID1
64	4299	AFF1
65	4331	MNAT1/SLC38A6
66	4543	MTNR1A
67	4684	NCAM1
68	4724	NDUFS4
69	4781	NFIB
70	4790	NFKB1
71	4803	NGF
72	4849	CNOT3
73	4892	NRAP
74	4898	NRD1
75	4948	OCA2
76	4999	ORC2
77	5071	PARK2
78	5101	PCDH9
79	5126	PCSK2
80	5139	PDE3A
81	5273	SERPINB10
82	5380	PMS2L2
83	5432	POLR2C/COQ9
84	5570	PKIB
85	5587	PRKD1
86	5592	PRKG1
87	5593	PRKG2
88	5646	PRSS3
89	5649	RELN
90	5737	PTGFR
91	5783	PTPN13
92	5789	PTPRD
93	5797	PTPRM
94	5801	PTPRR
95	5867	RAB4A/SPHAR
96	5891	MOK
97	5991	RFX3
98	6091	ROBO1
99	6196	RPS6KA2
100	6332	SCN7A
101	6342	SCP2
102	6368	CCL23
103	6444	SGCD
104	6450	SH3BGR
105	6546	SLC8A1
106	6555	SLC10A2
107	6557	SLC12A1/CTXN2
108	6638	GXL 1337704
109	6699	SPRR1B
110	6925	TCF4
111	6926	TBX3
112	7060	THBS4
113	7113	TMPRSS2
114	7223	TRPC4
115	7225	TRPC6
116	7227	TRPS1
117	7325	UBE2E2
118	7404	UTY
119	7405	UVRAG
120	7411	VBP1
121	7629	ZNF76
122	7697	ZNF138
123	7813	EVI5
124	7957	EPM2A
125	8170	SLC14A2
126	8301	PICALM
127	8405	SPOP
128	8453	CUL2
129	8491	MAP4K3
130	8505	PARG
131	8546	AP3B1
132	8549	LGR5
133	8621	CDK13
134	8654	PDE5A
135	8667	EIF3H
136	8809	IL18R1/IL1RL1
137	8826	IQGAP1
138	8895	CPNE3
139	8899	PRPF4B
140	8925	HERC1
141	8945	BTRC
142	9147	NEMF
143	9348	NDST3
144	9353	SLIT2
145	9365	KL
146	9369	NRXN3
147	9378	NRXN1
148	9451	EIF2AK3
149	9456	HOMER1
150	9459	ARHGEF6
151	9472	AKAP6
152	9515	STXBP5L
153	9517	SPTLC2
154	9569	GTF2IRD1
155	9666	DZIP3
156	9712	USP6NL
157	9734	HDAC9
158	9786	KIAA0586
159	9805	SCRN1
160	9832	JAKMIP2
161	9863	MAGI2
162	9892	SNAP91
163	9910	RABGAP1L
164	9933	KIAA0020
165	9988	DMTF1
166	10082	GPC6
167	10144	FAM13A
168	10178	ODZ1
169	10274	STAG1
170	10283	CWC27
171	10395	DLC1
172	10413	YAP1
173	10428	CFDP1
174	10450	PPIE
175	10464	PIBF1
176	10635	RAD51AP1
177	10699	CORIN
178	10955	SERINC3
179	11036	GXL 693729
180	11046	SLC35D2
181	11112	HIBADH
182	11124	FAF1
183	11141	IL1RAPL1
184	11254	SLC6A14
185	11259	FILIP1L
186	11281	POU6F2
187	11329	STK38
188	22829	NLGN4Y
189	22841	RAB11FIP2
190	22862	FNDC3A
191	22871	NLGN1
192	22891	ZNF365
193	22920	KIFAP3
194	22986	SORCS3
195	23022	PALLD
196	23026	MYO16
197	23040	MYT1L
198	23063	WAPAL
199	23116	FAM179B
200	23122	CLASP2
201	23236	PLCB1
202	23266	LPHN2
203	23271	CAMSAP2
204	23284	LPHN3
205	23362	PSD3
206	23369	PUM2
207	23635	SSBP2
208	23670	TMEM2
209	23768	FLRT2
210	25827	FBXL2
211	25890	ABI3BP
212	26009	ZZZ3
213	26011	ODZ4
214	26033	ATRNL1
215	26035	GLCE
216	26040	SETBP1
217	26154	ABCA12
218	26254	OPTC
219	26280	IL1RAPL2
220	26289	AK5
221	26575	RGS17
222	27067	GXL_1237600
223	27255	CNTN6
224	27327	TNRC6A
225	27347	STK39
226	27436	EML4
227	29117	BRD7
228	29119	CTNNA3
229	29789	OLA1
230	29880	ALG5
231	29953	TRHDE/TPH2
232	29969	MDFIC
233	30010	NXPH1
234	30817	EMR2
235	30820	KCNIP1
236	50802	IGK@
237	50859	SPOCK3
238	50865	HEBP1
239	51022	GLRX2
240	51071	DERA
241	51088	KLHL5
242	51106	TFB1M
243	51144	GXL 1223060
244	51163	DBR1
245	51176	LEF1
246	51196	PLCE1
247	51281	ANKMY1
248	51411	BIN2
249	51430	C1orf9
250	51474	LIMA1

251	51652	GXL_744663
252	51668	HSPB11
253	51773	RSF1
254	53344	CHIC1
255	53942	CNTN5
256	54221	SNTG2
257	54530	DENND1B
258	54715	RBFOX1
259	54765	TRIM44
260	54768	HYDIN
261	54886	LPPR1
262	54893	MTMR10
263	54901	CDKAL1
264	54916	C14orf101
265	54918	CMTM6
266	54927	CHCHD3
267	54968	TMEM70
268	55016	41334
269	55105	GPATCH2
270	55120	FANCL
271	55227	LRRC1
272	55269	PSPC1
273	55294	FBXW7
274	55296	TBC1D19
275	55582	KIF27
276	55714	ODZ3
277	55727	BTBD7
278	55733	HHAT
279	55753	OGDHL
280	55788	LMBRD1
281	56164	STK31
282	56521	DNAJC12
283	56829	ZC3HAV1
284	56884	FSTL5
285	56890	MDM1
286	56899	ANKS1B
287	56907	SPIRE1
288	57037	ANKMY2
289	57099	AVEN
290	57161	PELI2
291	57223	SMEK2
292	57512	GPR158
293	57554	LRRC7
294	57568	SIPA1L2
295	57575	PCDH10
296	57626	KLHL1
297	57628	DPP10
298	57678	GPAM
299	57687	VAT1L
300	57700	FAM160B1
301	58155	PTBP2
302	60560	NAA35
303	60678	EEFSEC
304	63901	FAM111A
305	64062	RBM26
306	64067	NPAS3
307	64122	FN3K
308	64123	ELTD1
309	64225	ATL2
310	64753	CCDC136
311	64784	CRTC3
312	64864	RFX7
313	65008	MRPL1
314	65217	PCDH15
315	79068	FTO
316	79591	C10orf76
317	79618	HMBOX1
318	79659	DYNC2H1
319	79699	ZYG11B
320	79776	ZFHX4
321	79800	ALS2CR8
322	79817	MOB3B
323	79839	CCDC102B
324	79848	CSPP1
325	79863	RBFA
326	79925	SPEF2
327	79971	WLS
328	79977	GRHL2
329	79993	ELOVL7
330	80135	RPF1
331	80144	FRAS1
332	80310	PDGFD
333	80333	KCNIP4
334	81537	SGPP1
335	81550	TDRD3
336	81617	CAB39L
337	83544	DNAL1
338	83641	FAM107B
339	83988	NCALD
340	84083	ZRANB3
341	84141	FAM176A
342	84187	TMEM164
343	84303	CHCHD6
344	84305	WIBG
345	84319	C3orf26
346	84530	SRRM4
347	84871	AGBL4
348	84898	PLXDC2
349	84899	TMTC4
350	85413	SLC22A16
351	89978	ATPBD4
352	90102	PHLDB2,PLCXD2
353	90362	FAM110B
354	91351	DDX60L
355	91452	ACBD5
356	91942	NDUFAF2
357	93035	PKHD1L1
358	93164	HTR7P1
359	93663	ARHGAP18/TMEM244
360	114788	CSMD3
361	114815	SORCS1
362	114882	OSBPL8
363	115825	WDFY2
364	116496	FAM129A
365	116931	MED12L
366	118426	LOH12CR1
367	121512	FGD4
368	121601	ANO4
369	123624	AGBL1
370	123722	FSD2
371	128611	ZNF831
372	131118	DNAJC19
373	135228	CD109
374	138046	RALYL
375	140545	RNF32
376	144402	CPNE8
377	144766	LINC00355
378	144983	HNRNPA1L2
379	151531	UPP2
380	151651	EFHB
381	151742	PPM1L
382	152330	CNTN4
383	153129	SLC38A9
384	153579	BTNL9
385	154215	NKAIN2
386	158038	LINGO2
387	158326	FREM1
388	160335	TMTC2
389	161357	MDGA2
390	163223	ZNF99/ZNF676
391	165215	FAM171B
392	166378	SPATA5
393	168975	CNBD1
394	170506	DHX36
395	170591	S100Z
396	196528	ARID2
397	199777	ZNF626
398	200844	C3orf67
399	202559	KHDRBS2
400	203100	HTRA4
401	204962	SLC44A5
402	220164	DOK6
403	220929	ZNF438
404	221322	C6orf170
405	221336	BEND6
406	222234	FAM185A
407	223117	SEMA3D
408	253714	MMS22L
409	255119	FGF5/C4orf22
410	255631	COL24A1
411	256076	COL6A5
412	256536	TCERG1L
413	257194	NEGR1
414	284222	PIEZO2
415	285335	SLC9C1
416	285780	LY86-AS1
417	286187	COPS5/PPP1R42
418	286411	RP1-177G6.2
419	338645	LUZP2
420	339010	POTEB
421	339479	FAM5C
422	342574	KRT27
423	344148	NCKAP5
424	348980	HCN1
425	374393	FAM111B
426	374864	C18orf34
427	375449	MAST4
428	375612	LHFPL3
429	389170	LEKR1
430	399761	BMS1P5
431	400509	SNX29P1
432	400800	FLJ43585
433	400945	FLJ41481
434	401050	LOC100288428
435	401145	FAM190A
436	401541	CENPP
437	404033	GXL_1222485
438	439936	C5orf17
439	440153	OR11H12
440	441369	FLJ46284
441	441666	LOC441666
442	442028	LOC442028
443	442213	PTCHD4
444	442319	ZNF727
445	644444	TMEM30C
446	647946	LOC647946
447	653238	GTF2H2B
448	653252	GXL_1336332
449	728411	GUSBP1
450	728621	CCDC30
451	100127889	ENTPD1
452	100128590	LOC100128590
